# Pain, Function, and Elastosonographic Assessment After Shockwave Therapy in Non-Calcific Supraspinatus Tendinopathy: A Retrospective Observational Study

**DOI:** 10.3390/jfmk10010039

**Published:** 2025-01-21

**Authors:** Gabriele Santilli, Antonello Ciccarelli, Milvia Martino, Patrizia Pacini, Francesco Agostini, Andrea Bernetti, Luca Giuliani, Giovanni Del Gaudio, Massimiliano Mangone, Vincenzo Colonna, Mario Vetrano, Maria Chiara Vulpiani, Giulia Stella, Samanta Taurone, Federico Vigevano, Vito Cantisani, Marco Paoloni, Pietro Fiore, Francesca Gimigliano

**Affiliations:** 1 Department of Movement, Human and Health Sciences, Division of Health Sciences, University of Rome “Foro Italico”, 00135 Rome, Italy; 2 Department of Radiological Sciences, Oncology and Pathology, Policlinico Umberto I, Sapienza University of Rome, 00161 Rome, Italy; 3 Department of Anatomical and Histological Sciences, Legal Medicine and Orthopedics, Sapienza University, 00185 Rome, Italy; 4 Department of Biological and Environmental Science and Technologies, University of Salento, 73100 Lecce, Italy; 5 Physical Medicine and Rehabilitation Unit, Sant’Andrea Hospital, Sapienza University of Rome, 00189 Rome, Italy; 6 Neurorehabilitation and Adapted Physical Activity Day Hospital, Bambino Gesù Children’s Hospital, IRCCS, 00165 Rome, Italy; 7 Paediatric Neurorehabilitation Department, IRCCS San Raffaele, 00163 Rome, Italy; 8 Neurorehabilitation Unit, Institute of Bari, Istituti Clinici Scientifici Maugeri IRCCS, 70124 Bari, Italy; 9 Department of Physical and Mental Health and Preventive Medicine, University of Campania “Luigi Vanvitelli”, 80100 Naples, Italy

**Keywords:** supraspinatus tendinopathy, extracorporeal shockwave therapy, shear wave elastography, tendon thickness, SWE velocity, rotator cuff disorders, pain assessment, functional recovery, shoulder, chronic tendinopathy

## Abstract

**Background:** Non-calcific supraspinatus tendinopathy (SNCCT) is a frequent cause of shoulder pain, often associated with functional impairment and reduced quality of life. Recent advancements in diagnostic imaging, including shear wave elastography (SWE), provide quantitative data on tendon stiffness and thickness, facilitating more precise evaluations. Extracorporeal shockwave therapy (ESWT) has emerged as a minimally invasive and effective treatment for SNCCT, but its effects on tendon properties measured through SWE require further investigation. **Objective:** This retrospective observational study aimed to evaluate the impact of ESWT on supraspinatus tendon characteristics in patients with SNCCT by assessing tendon thickness, SWE velocity, and clinical outcomes. Methods: This observational study enrolled 39 patients with SNCCT, aged 30–75 years, who received three ESWT sessions over 3 weeks. The intervention was delivered using a Modulith SLK system at an energy level of 0.20 mJ/mm^2^ with 2400 pulses per session. SWE and conventional ultrasound were used to measure tendon thickness and SWEv at baseline (T0) and 6 months post-treatment (T1). Clinical outcomes were assessed using the Visual Analog Scale (VAS), Constant and Murley Score (CMS), and modified Roles and Maudsley scale. Data were analyzed using paired *t*-tests and correlation analyses. **Results:** At baseline, affected tendons exhibited increased thickness (7.5 ± 0.9 mm) and reduced SWEv (3.1 ± 0.7 m/s) compared to healthy tendons (4.5 ± 0.7 mm and 6.9 ± 1 m/s, respectively; *p* < 0.05). Six months after ESWT, tendon thickness decreased significantly (6.2 ± 0.9 mm, *p* < 0.05), and SWEv increased (5.7 ± 1.8 m/s, *p* < 0.05), indicating improved elasticity. Clinical outcomes improved significantly, with the VAS scores decreasing from 6.5 ± 1.4 to 3.2 ± 2.1, the CMS score rising from 59.1 ± 17.3 to 78.2 ± 17.7, and the modified Roles and Maudsley scale improving from 2.3 ± 0.6 to 1.5 ± 0.8 (*p* < 0.05 for all). SWEv positively correlated with the CMS (r = 0.4) and negatively with the VAS and the modified Roles and Maudsley scale (r = −0.6 and r = −0.5, respectively). **Conclusions:** ESWT significantly reduces tendon thickness and enhances elasticity, correlating with improvements in pain and functional scores. SWE proved to be a reliable method for monitoring structural and clinical changes in SNCCT. Further research, including randomized controlled trials, is recommended to confirm these findings and explore longer-term outcomes.

## 1. Introduction

The supraspinatus tendon is frequently implicated in shoulder pain and is commonly affected by inflammatory conditions that lead to joint dysfunction [1,2,3,4]. This inflammation is typically associated with pain, resulting in reduced functionality and performance [5,6,7]. Rotator cuff tendinopathy has a multifactorial origin involving both intrinsic and extrinsic factors [8]. The diagnosis of supraspinatus-related issues is based on a combination of patient history [9] and physical examination, with reliable tests including the drop arm test, empty can test, full can test, and the shoulder lift-off sign [10]. To diagnose musculoskeletal disorders, ultrasound and magnetic resonance imaging (MRI) are valuable [11,12,13,14,15,16,17,18,19,20], in particular for identifying rotator cuff (RC) disorders [21]. In recent years, elastosonography, and specifically shear wave elastography (SWE), has begun to be used to evaluate rotator cuff pathologies, with a particular focus on the supraspinatus tendon [22,23]. To assess the pain and functional status of patients with non-calcific supraspinatus tendinopathy (SNCCT), evaluation scales such as the Visual Analog Scale (VAS), the Constant and Murley Score (CMS), and the modified Roles and Maudsley scale are commonly employed, as they are widely used to measure shoulder pain, functional capacity, and treatment outcomes [24,25]. Additionally, a modified Roles and Maudsley scale was utilized to assess functional outcomes more comprehensively by adapting it to better reflect subtle clinical changes in SNCCT patients [26]. The VAS, CMS and modified Roles and Maudsley scale are not only assessment tools but also prognostic predictors [27].

Treatment options vary depending on the severity of the condition and whether tendon tears are present. In recent years, the treatment of tendinopathies, particularly rotator cuff tendinopathy, has been extensively explored through various methods. These include rehabilitative exercises, physical therapy, non-steroidal anti-inflammatory drugs, local injections, prolotherapy, platelet-rich plasma, hyaluronic acid, botulinum toxin, kinesiotaping, laser and thermal therapy, extracorporeal shockwave therapy (ESWT), and surgical interventions followed by rehabilitation [28,29,30,31,32,33,34,35]. In recent years, ESWT has gained recognition as an effective and safe approach to upper limb pain and managing supraspinatus tendinopathy and other pathologies related to rotator cuff tears [36,37,38,39]. The increasing popularity of ESWT is attributed to several factors: its high clinical success rate [40], the convenience of minimal treatment sessions, brief therapy durations, cost efficiency, improved accessibility, and the lack of significant activity restrictions during treatment. Despite treatment, some patients may experience persistent symptoms or recurrence of supraspinatus tendinopathy, negatively impacting their quality of life [41].

Being able to carry out a more in-depth evaluation that is not subjective but based on diagnostic findings could be crucial in guiding clinical decision-making. With this perspective, our study assessed tendon stiffness using elastosonography, along with tendon thickness, to provide a more comprehensive evaluation of these patients.

Compared to previous studies, this approach integrates SWE to quantitatively evaluate tendon elasticity and thickness alongside clinical scales such as the CMS, the modified Roles and Maudsley scale, and the VAS, providing a precise and objective correlation between structural changes and functional improvements. This combined methodology enhances our understanding of ESWT’s effects on tendon pathology, addressing current limitations in the literature.

This study is a retrospective observational study aimed to evaluate the characteristics of the supraspinatus tendon in patients with chronic SNCCT before and after ESWT treatment. This evaluation incorporated clinical parameters, conventional gray-scale imaging, and ultrasound elastography using SWE techniques.

## 2. Materials and Methods

### 2.1. Study Design and Population

This observational study follows the ethics of the Helsinki Declaration, approved by La Sapienza University’s Institutional Review Board (Prot. 0000/2024—Approval Date: 14 February 2024). The STROBE checklist for observational studies was followed for this study. All patients signed informed consent forms, and their data were anonymized. Participants provided written consent prior to this study, including explicit agreement on the processing of personal data for research purposes, with anonymization measures ensuring privacy protection. The data of patients who received therapy were extracted from a pre-existing dataset. SNCCT diagnosis was established through clinical symptoms, physical examinations, and imaging studies. Eligibility was determined for all patients meeting the following selection criteria: (1) age between 30 and 75 years. This age range was selected as it represents a demographic group commonly analyzed in SNCCT and frequently treated with ESWT in clinical practice [27]. Since biomechanical differences related to age (such as tendon elasticity and healing response) may introduce variability, a specific age stratification was performed, based on a previous study [42], to reduce potential biases and ensure that baseline clinical characteristics and tendon properties were comparable across the age groups, and no statistically significant differences were found between the age groups at the baseline; (2) pain localized to the lateral aspect of the shoulder, worsening with overhead activities; (3) localized shoulder pain; (4) symptoms persisting for at least 3 months; (5) inclusion of both male and female patients; (6) reduced shoulder range of motion; and (7) a positive rotator cuff tendinopathy test.

**Figure 1:**
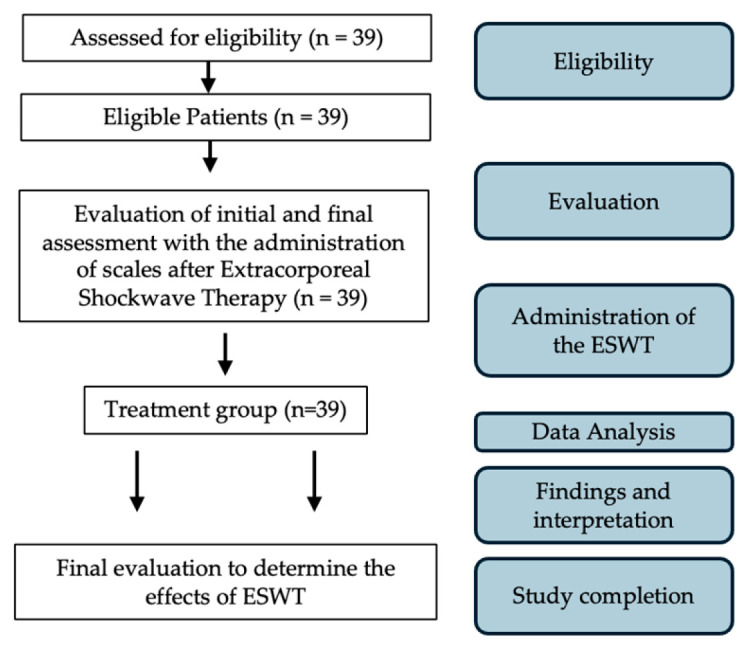
Flow diagram of this study.

Exclusion criteria included: (1) significant atrophy or weakness of any shoulder girdle muscle; (2) a history of shoulder surgery; (3) recent use of corticosteroids or nerve blocks; (4) presence of a tumor in the treatment area; (5) pregnancy; and (6) coagulation disorders. All eligible patients completed a demographic and clinical questionnaire that assessed age, gender, and scales administered at T0 and at T1 after 6 months from ESWT were the modified Roles and Maudsley scale, VAS score, and CMS and the ultrasonographic evaluation of the thickness and the SWEv of the supraspinatus tendon at baseline and at T1. A flow diagram of this study is shown in Figure 1.

### 2.2. Intervention

**Figure 2:**
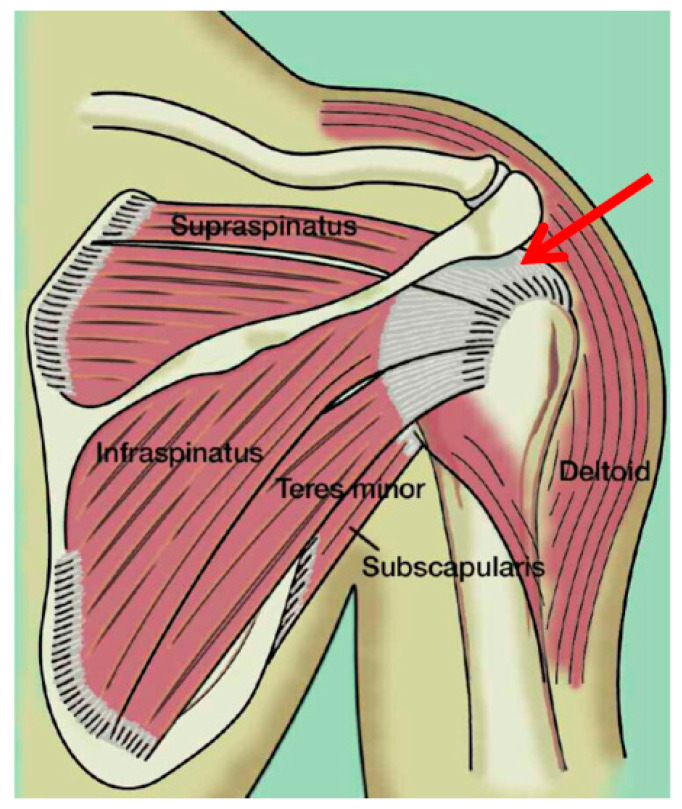
Schematic representations of the tendon where ESWT was administrated (arrow red).

The study protocol used was in line with the current state of the art in treating supraspinatus tendon with ESWT performed by the principal authors with the Modulith SLK system (Storz Medical, Tagerwilen, Switzerland), with an electromagnetic extracorporeal shockwave generator equipped with an in-line ultrasound positioning system on the target zone at 2 cm from the insertion point of the supraspinatus tendon on the greater tuberosity (Figure 2). All treatments were conducted without local anesthesia. Participants received ESWT while lying supine on a bed, with the affected arm positioned in adduction, the elbow flexed at 90 degrees, and the hand placed under the ipsilateral gluteus. The ESWT protocol consisted of 0.20 mJ/mm^2^ energy, 2400 pulses, and a frequency of 6 Hz, administered once a week for 3 consecutive weeks [43]. Patients were treated with three sessions of ESWT once a week. Clinical and ultrasound evaluations were performed at the baseline (T0) when patients underwent the first ESWT treatment and at 6 months (T1) after the end of ESWT treatment. The VAS, CMS, and modified Roles and Maudsley scale were administered before treatment and at 6 months after the end of the ESWT. All patients completed a 24-week follow-up (T1).

#### 2.2.1. Outcomes Ultrasound Evaluation

The outcome data were collected by a physiatrist. The Visual Analog Scale (VAS) comprises a 100 mm horizontal line, with “no pain” denoted at the left end (score: 0) and “pain as severe as possible” at the right end (score: 10). Patients were instructed to place a hatch mark on the line corresponding to their current pain level during their most painful movement. The VAS score was subsequently determined by measuring the distance in millimeters between the left endpoint and the patient’s mark [24].

To assess symptom severity and patient functionality, the CMS score questionnaire translated and validated for Italian was applied [44]. This is a 100-item instrument used to provide a clinical assessment of the shoulder in terms of pain severity (maximum score of 15), the ability of the patient to perform activities of daily living (maximum score of 20), active range of motion (maximum score of 30), and shoulder strength (maximum score of 25). On this scale, a score of 0 indicates that the patient has intense pain and is unable to perform activities of daily living using the shoulder in question. The maximum score of 100 indicates that the patient is pain-free and able to execute all the activities of daily living. This instrument is a compound score containing four subscales: two self-reported subscales and two scales administered by an external rater. Normal values are between 70 and 90 points [45].

The modified Roles and Maudsley scale was used to evaluate the patient’s pain in relation to normal daily activities. The modified Roles and Maudsley scale of 1 represented excellent quality of life (i.e., no symptoms; unlimited walking ability without pain; patient satisfied with the treatment outcome [when assessed after RSWT or placebo]), 2 represented good quality of life (i.e., ability to walk more than 1 hour without pain; symptoms substantially decreased after treatment; patient satisfied with the treatment outcome); 3 indicated acceptable quality of life (i.e., inability to walk more than one hour without pain; symptoms somewhat better and pain more tolerable than before treatment; patient slightly satisfied with the treatment outcome); 4 indicated poor quality of life (i.e., inability to walk without severe pain; symptoms not better or even worse after treatment; patient not satisfied with the treatment outcome) [26].

#### 2.2.2. Ultrasound Evaluation

**Figure 3:**
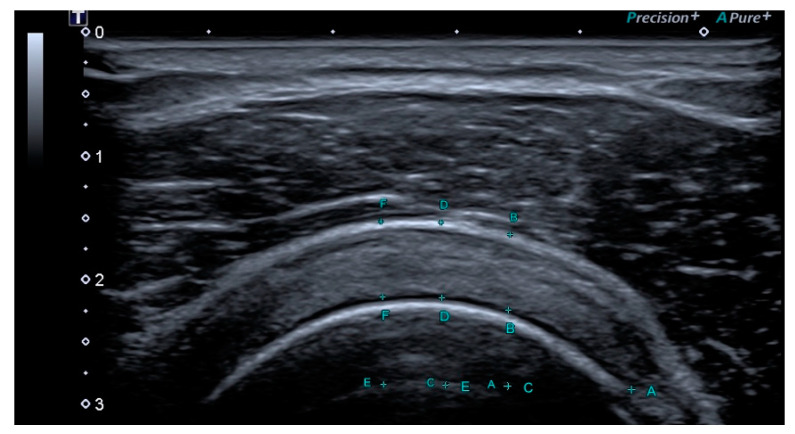
Supraspinatus tendon thickness in the short-axis (transverse) image; the average of three tendon thickness measures taken at 10, 15, and 20 mm lateral to the biceps tendon (#A).

All the examinations were performed by a musculoskeletal physician with 10 years of experience in imaging and more than 24 months of experience in ultrasound elastography, using a Toshiba Aplio 500 machine (Canon Medical System Europe BV) equipped with a multi-frequency 5–18 MHz linear probe. The patients were positioned supine to ensure a consistent and relaxed posture. The shoulder was placed in a neutral position with slight external rotation, and participants were instructed to rest their involved hand on the ipsilateral posterior hip, with the humerus in extension. The ultrasound transducer was placed on the anterior aspect of the shoulder, perpendicular to the supraspinatus tendon and just anterior to the anterior–lateral margin of the acromion to capture both the supraspinatus tendon and long head of the biceps tendon laterally in short axis. The transducer was tilted medial/lateral to ensure the biceps tendon was visualized and maximum tendon thickness was obtained in short axis. Three positions along the tendon were measured for thickness in millimeters (mm) at 10, 15, and 20 mm lateral to the reference point of the lateral point of hyperechogenicity of the biceps tendon (Figure 3). The average of three measures was taken due to the irregular thickness of the tendon [46].

**Figure 4:**
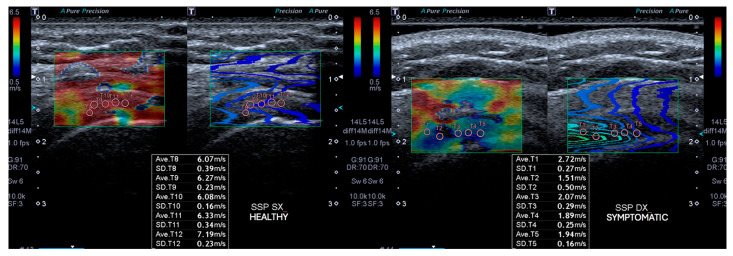
ROI measurements on shear wave elastography velocity (SWEv) images in 55-year-old woman with chronic non-calcific supraspinatus tendinopathy (SNCCT).

SWE was performed immediately following the B-mode ultrasound examination using a system equipped with shear wave elastography capabilities. The shear wave elastography velocity (SWEv) was recorded in meters per second (m/s). The transducer was oriented in the longitudinal plane of the supraspinatus tendon, ensuring clear visualization of the tendon fibers near the insertion site. Five circular regions of interest (ROIs), each with a diameter of 3 mm [47], were placed within the insertional supraspinatus tendon. The ROIs were evenly distributed across the tendon tissue to capture representative measurements. Particular attention was given to avoiding areas with visible artifacts or irregularities on the propagation map. For each ROI, the SWE system automatically calculated the SWEv, and the mean value of these five measurements was recorded as the elasticity value for the supraspinatus tendon, as shown in Figure 4.

### 2.3. Statistical Analysis

A power analysis was conducted using G*Power (v.3.1.9.2, developed by Franz Faul and colleagues at the University of Kiel, Germany) to ensure an adequate sample size for detecting a meaningful effect. Drawing on the study by Riaz S et al. [48], we aimed for a statistical power of 90% to identify a 1.5-point difference in the VAS score, employing a two-tailed *t*-test with Bonferroni corrections. The precision level was determined with a standard deviation (SD) of 1.5 points [49]. A confidence level of 95% (α = 0.05) and an effect size of 0.93 were used to estimate the magnitude of practically significant differences. Based on these parameters, a minimum sample size of 21 participants was calculated as sufficient. In our study, we included 39 patients, which exceeds the required minimum sample size to detect statistically significant differences. While we acknowledge that a relatively small sample size may limit the generalizability of our findings, it is important to emphasize the exploratory nature of this study and its aim to provide preliminary insights into the effects of ESWT on SNCCT.

All statistical analyses were performed using SPSS version 28 (IBM Corp, Armonk, NY, USA). The normality of the data was confirmed using the Shapiro–Wilk test. Continuous variables were reported as means ± standard deviations (SDs), and categorical variables were presented as frequencies and percentages. Descriptive statistics (mean and standard deviations) were used to describe the characteristics of the outcome variables before and after intervention using paired samples t-tests to provide an understanding of the data.

Finally, to verify the correlation of the ultrasonographic outcomes with clinical scale outcomes, correlational analyses were performed. Bivariate correlations were performed to assess the relationships between variables; specifically, Pearson’s correlation was applied for continuous variables. A cut-off for correlations was considered as follows: values between 0.3 and 0.7 indicated a moderate positive linear relationship, while values between −0.3 and −0.7 indicated a moderate negative linear relationship [50].

## 3. Results

### 3.1. Patient Demographic and Clinical Characteristics

Between February 2022 and September 2024, 39 eligible patients with chronic SNCCT were evaluated and accepted to participate. The baseline demographic and clinical characteristics of patients are reported in Table 1.

### 3.2. Outcome Measures Results

At baseline, the affected side showed a thickened supraspinatus tendon compared with the healthy side (7.5 ± 0.9 vs. 4.5 ± 0.7 mm) (*p* < 0.05). Table 2 shows the B-mode and elastographic results at baseline and after ESWT treatment at T1. Six months after ESWT, the thickness of the symptomatic side decreased from the baseline (7.5 ± 0.9 vs. 6.2 ± 0.9 mm) (*p* < 0.05). Six months after ESWT, the affected side showed a thickened supraspinatus tendon compared with the healthy side (5.2 ± 1.3 vs. 4.5 ± 0.7 mm) (*p* < 0.05).

At baseline, the affected side showed lower supraspinatus tendon SWEv compared with the healthy side (3.1 ± 0.7 vs. 6.9 ± 1 mm) (*p* < 0.05). Six months after ESWT, the SWEv of the symptomatic side increased from the baseline (3.1 ± 0.7 vs. 5.7 ± 1.8 mm) (*p* < 0.05). Six months after ESWT, the affected side showed lower supraspinatus tendon SWEv compared with the healthy side (5.7 ± 1.8 vs. 6.9 ± 1 mm) (*p* < 0.05).

The baseline average for the VAS, CMS and modified Roles and Maudsley scale were, respectively, 6.5 points with ± 1.4 SD, 59.1 points with ± 17.3 points SD, and 2.3 points with ± 0.6 SD (Table 3). At the last follow-up at T1, the VAS, CMS and the modified Roles and Maudsley scale had an average of, respectively, 3.2 points with ± 2.1 SD, 78.2 points with ± 17.7 points SD, and 1.5 points with ± 0.8 SD.

To evaluate whether the elastographic technique yields different results compared to functional outcome measures, a bivariate Pearson correlation was performed. A significant statistical negative correlation was found between SWEv and the VAS score at T1 (r = −0.6; *p* < 0.05) and between SWEv and the modified Roles and Maudsley scale at T1 (r = −0.5; *p* < 0.05), and a significant statistical positive correlation was found between SWEv and the CMS score at T1 (r = 0.4; *p* < 0.05).

A significant statistical positive correlation was found between thickness and the VAS score at T1 (r = −0.65; *p* < 0.05) and between SWEv and the modified Roles and Maudsley scale score at T1 (r = −0.6; *p* < 0.05), and a significant statistical negative correlation was found between thickness and the CMS score at T1 (r = 0.5; *p* < 0.05).

### 3.3. Safety

Throughout this study, participants did not experience or report any significant adverse effects related to the therapies.

## 4. Discussion

In our study, symptomatic supraspinatus tendons demonstrated significantly lower SWEv values compared to asymptomatic ones, indicating that they were “softer” or “less elastic”. Regarding elastography values, our findings for the supraspinatus tendon align with previously reported data for the Achilles tendon [51] and the insertional region of the supraspinatus tendon [52]. This observation is further supported by our results regarding an increased thickness of symptomatic tendons compared to their healthy contralateral counterparts, which predominantly exhibited an edematous appearance. In contrast, the asymptomatic, healthy contralateral tendons were generally thinner. These findings suggest the presence of less rigid, edematous tissue in patients with supraspinatus tendinopathy, aligning with previously reported data in the literature [53,54,55]. Therefore, our observation that supraspinatus tendinopathy is characterized by increased tendon thickness and reduced SWE values aligns with previous findings regarding the common extensor tendon. Specifically, studies on lateral epicondylitis have demonstrated a similar pattern, with the affected side showing greater thickness and lower SWE values compared to the healthy side [56]. These observations reinforce the validity of SWE as a diagnostic and monitoring tool across different anatomical regions. SWEv effectively describes the structural alterations in the supraspinatus tendons caused by tendinopathy, consistently showing lower values in affected tendons compared to healthy asymptomatic ones [52]. This result may be interpreted as reduced elasticity on the affected side, likely due to degenerative processes such as collagen breakdown, fibroblastic hypertrophy, matrix degradation, and vascular ingrowth, as observed in the connective tissue of cadaveric surgical biopsies [57,58,59]. These changes reflect the potential of ESWT to induce structural and functional recovery in tendons affected by chronic tendinopathy.

The SWEv values increased from baseline, highlighting its utility in detecting improvements in supraspinatus tendons, which is in agreement with conventional ultrasound findings. In past studies, a reduction in the supraspinatus tendon thickness, along with clinical symptom improvement, was reported following the therapies [60,61]. In recent years, multiple studies have established that SWE is an effective method for distinguishing diseased tendons from healthy ones, with diseased tendons exhibiting significantly lower stiffness [42,62,63,64]. Beyond merely identifying “healthy” versus “diseased” tendons, SWE provides valuable diagnostic insights by quantitatively assessing the extent of tendon impairment [65]. These quantitative data, which complement conventional B-mode ultrasound findings, make SWE a dependable tool for visualizing clinically significant tendon damage.

Our findings have practical implications for the clinical management of supraspinatus tendinopathy. SWEv and tendon thickness emerge as useful biomarkers for monitoring disease progression and therapeutic response. These parameters provide objective insights into tendon health and can complement traditional clinical evaluations such as the VAS and modified Roles and Maudsley scale.

In our study, 6 months after ESWT therapy, we observed improvements in pain assessed via the VAS and function evaluated through the modified Roles and Maudsley scale and CMS score. These improvements were correlated to both an increase in SWEv and a reduction in the thickness of the supraspinatus tendons. The potential influence of the placebo effect cannot be entirely excluded, as improvements in pain and function may partially stem from patient expectations and therapeutic interaction [66]. However, it is important to note that ultrasound evaluations, such as those using SWE, are objective measures and are not influenced by placebo effects. These findings may be associated with the synthesis of new collagen and the subsequent restoration of connective tissue characteristics following ESWT treatment, as described by Vetrano et al. [67]. Additionally, the effects of ESWT on tissue—activating a neovascularization process [68], amplifying growth factor activity and protein synthesis to stimulate collagen production and tissue remodeling [69]—suggest that stem cells in the tendon may have been influenced by this therapy, triggering beneficial effects, as highlighted by previous research [70,71].

The limitations and constraints of SWE have been highlighted [72], particularly when assessing tendon structure, emphasizing the need for a systematic and structured approach. However, some studies have demonstrated that SWE outperforms conventional ultrasound in terms of specificity, sensitivity, and accuracy for diagnosing tendinopathy [72].

## 5. Strengths and Weaknesses of this Study

Despite these promising results, several limitations must be acknowledged. This study’s retrospective design may introduce selection bias, as only patients who completed the full course of treatment were included in the analysis. Furthermore, the lack of a control group limits the ability to definitively attribute the observed improvements to the interventions alone. Future randomized controlled trials and comparisons with healthy individuals would be necessary to confirm these findings and establish a causal relationship. Future studies with larger cohorts [73] are warranted to confirm and further validate our results.

Limits of this preliminary study are the use of contralateral healthy side tendons as a control, the small sample size, and the fact that despite the long-term effects of ESWT, we did not follow these patients long enough to evaluate the recurrence rate. Further longer-term follow-up studies are mandatory. Selection bias is inherent in the retrospective design, as this study included only patients who completed the full course of treatment, potentially leading to an overestimation of the observed effects. The external validity is diminished as our sample only encompasses a particular geographical region [74]. Moreover, with the advancement of artificial intelligence and machine learning methods, future studies should explore this aspect, especially in physical and rehabilitation medicine and tendinopathies, as previously developed studies [75,76].

Overall, this study contributes to the growing body of evidence supporting the use of ESWT for SNCCT. Future studies should focus on long-term outcomes, the potential for relapse, and the exploration of other adjunctive therapies to enhance treatment effectiveness.

## 6. Conclusions

In conclusion, SWE seems to be a useful elastographic technique to assess supraspinatus tendon elasticity and its alteration in chronic non-calcific supraspinatus tendinopathy. Furthermore, on the basis of the correlation with pain and functional scales, this technique appears useful in addition to conventional ultrasound for monitoring the efficacy of treatment, providing quantitative data. However, further prospective evaluations on a larger population and randomized controlled trials are warranted to confirm these promising results.

## Figures and Tables

**Table 1:** Comparison of baseline variables and outcomes measures before and after treatment. Values are reported as mean ± standard deviation for continuous variables and as distribution for categoric variables.

Variable	Value
Age (years)	61 ± 12.8
Gender (Male/Female)	17/22
VAS T0	6.5 ± 1.4
VAS T1	3.2 ± 2.1
CMS T0	59.1% ± 17.3%
CMS T1	78.2% ± 17.7%
RM T0	2.3 ± 0.6
RM T1	1.5 ± 0.8
Thickness affected side T0	7.5 ± 0.9
Thickness affected side T1	5.3 ± 1.3
Thickness healthy side T0	4.5 ± 0.7
SWEv affected side T0	3 ± 0.7
SWEv affected side T1	5.7 ± 1.8
SWEv healthy side T0	6.9 ± 1

VAS: Visual Analog Scale; CMS: Constant and Murley score; RM: Roles and Maudsley scale; SWEv: shear wave elastography velocity (m/s).

**Table 2:** B-mode and elastographic evaluation at baseline and after ESWT treatment.

	Symptomatic SSP T0	Symptomatic SSP T1	95% C.I.	Healthy SSP T1	*p*-Value
Supraspinatus Thickness	7.5 ± 0.9	6.2 ± 0.9	−0.9 to −1.6	4.5 ± 0.7	<0.05
SWEv (m/s)	3.1 ± 0.7	5.7 ± 1.8	3.2 to 2.1	6.9 ± 1	<0.05

Data presented as median (range). T0: baseline, T1: 6 months after treatment. SSP: supraspinatus tendon; SWEv: shear wave elastography velocity.

**Table 3:** Mean and standard error of Visual Analog Scale (VAS) Scores, Roles and Maudsley (RM) scores and Constant and Murley Scores of patients with chronic non-calcific supraspinatus tendinopathy after treatment with extracorporeal shockwave therapy (ESWT).

	VAS Score	VAS 95% C.I.	Constant and Murley Score	CMSs 95% C.I.	Roles and Maudsley Scale	RM 95% C.I.
Baseline T0	6.5 ± 1.4	−2.4 to −3.7	59.1 ± 17.3	23.9 to 14.3	2.3 ± 0.6	−0.3 to −0.9
Follow-up T1	3.2 ± 2.1		78.2 ± 17.7		1.5 ± 0.8	

Data presented as median (range). T0: baseline, T1: 6 months after treatment. CMSs: Constant and Murley score; RM: Roles and Maudsley scale.

## Data Availability

The datasets used, and data analyzed during the current study will be made available upon reasonable request to the corresponding author (G.S.).

## References

[1] Varacallo M., El Bitar Y., Mair S.D. (2020). Rotator Cuff Syndrome.

[2] Spargoli G. (2018). Supraspinatus Tendon Pathomechanics: A Current Concepts Review. Int. J. Sports Phys. Ther..

[3] Merolla G., Singh S., Paladini P., Porcellini G. (2016). Calcific tendinitis of the rotator cuff: State of the art in diagnosis and treatment. J. Orthop. Traumatol..

[4] Chianca V., Albano D., Messina C., Midiri F., Mauri G., Aliprandi A., Catapano M., Pescatori L.C., Monaco C.G., Gitto S. (2018). Rotator cuff calcific tendinopathy: From diagnosis to treatment. Acta Bio Medica Atenei Parm..

[5] Kaux J.-F., Forthomme B., Le Goff C., Crielaard J.-M., Croisier J.-L. (2011). Current opinions on tendinopathy. J. Sports Sci. Med..

[6] Del Buono A., Battery L., Denaro V., Maccauro G., Maffulli N. (2011). Tendinopathy and Inflammation: Some Truths. Int. J. Immunopathol. Pharmacol..

[7] Riley G. (2008). Tendinopathy—From basic science to treatment. Nat. Clin. Pract. Rheumatol..

[8] Seitz A.L., McClure P.W., Finucane S., Boardman N.D., Michener L.A. (2011). Mechanisms of rotator cuff tendinopathy: Intrinsic, extrinsic, or both?. Clin. Biomech..

[9] Pissarra Â., Ribeiro L., Rodrigues S. (2024). Ultrasonographic Evaluation of the Patellar Tendon in Cyclists, Volleyball Players, and Non-Practitioners of Sports—The Influence of Gender, Age, Height, Dominant Limb, and Level of Physical Activity. J. Funct. Morphol. Kinesiol..

[10] Moreno-Fernandez J.M., Martinez-Martinez F., Santon-ja-Medina F. (2019). Inter-Observer Reliability of Physical Examination in the Painful Shoulder: Supraspinatus Tendinopathy. Arch. Sports Med..

[11] Zoga A.C., Kamel S.I., Hynes J.P., Kavanagh E.C., O’Connor P.J., Forster B.B. (2021). The Evolving Roles of MRI and Ultrasound in First-Line Imaging of Rotator Cuff Injuries. Am. J. Roentgenol..

[12] Pacini P., Martino M., Giuliani L., Santilli G., Agostini F., Del Gaudio G., Bernetti A., Mangone M., Paoloni M., Toscano M. (2023). Patello-Femoral Pain Syndrome: Magnetic Resonance Imaging versus Ultrasound. Diagnostics.

[13] Schreiner J., Scheicht D., Karakostas P., Recker F., Ziob J., Behning C., Preuss P., Brossart P., Schäfer V. (2023). Prevalence of joint, entheseal, tendon, and bursal findings in young, healthy individuals by musculoskeletal ultrasound. Scand. J. Rheumatol..

[14] Oh J.H., Rhee S.M., Park J.H., Lee K.J., Yoon J.Y., Jeon Y.D., Kim H.S. (2022). Quantitative magnetic resonance imaging assessment of the infraspinatus and teres minor in massive rotator cuff tear and its significance in clinical outcome after rotator cuff repair. J. Shoulder Elb. Surg..

[15] Pessali-Marques B., Burden A.M., Morse C.I., Onambélé-Pearson G.L. (2024). Musculoskeletal Morphology and Joint Flexibility-Associated Functional Characteristics across Three Time Points during the Menstrual Cycle in Female Contemporary Dancers. J. Funct. Morphol. Kinesiol..

[16] Ivanov I., Ranchev S., Stoychev S. (2024). Experimental Ultrasound Approach for Studying Knee Intra-Articular Femur–Tibia Movements under Different Loads. J. Funct. Morphol. Kinesiol..

[17] Yamada Y., Inui K., Okano T., Mandai K., Mamoto K., Koike T., Takeda S., Yamashita E., Yoshida Y., Tateishi C. (2021). Ultrasound assessment, unlike clinical assessment, reflects enthesitis in patients with psoriatic arthritis. Clin. Exp. Rheumatol..

[18] Lee Y.H., Yang J., Jeong H.-K., Suh J.-S. (2017). Assessment of the patellofemoral cartilage: Correlation of knee pain score with magnetic resonance cartilage grading and magnetization transfer ratio asymmetry of glycosaminoglycan chemical exchange saturation transfer. Magn. Reson. Imaging.

[19] Duran S., Cavusoglu M., Kocadal O., Sakman B. (2017). Association between trochlear morphology and chondromalacia patella: An MRI study. Clin. Imaging.

[20] Osawa Y., Arai Y., Oguma Y., Hirata T., Abe Y., Azuma K., Takayama M., Hirose N. (2017). Relationships of Muscle Echo Intensity with Walking Ability and Physical Activity in the Very Old Population. J. Aging Phys. Act..

[21] Roy J.-S., Braën C., Leblond J., Desmeules F., E Dionne C., MacDermid J.C., Bureau N.J., Frémont P. (2015). Diagnostic accuracy of ultrasonography, MRI and MR arthrography in the characterisation of rotator cuff disorders: A systematic review and meta-analysis. Br. J. Sports Med..

[22] Zhou J., Yang D.-B., Wang J., Li H.-Z., Wang Y.-C. (2020). Role of shear wave elastography in the evaluation of the treatment and prognosis of supraspinatus tendinitis. World J. Clin. Cases.

[23] Itoigawa Y., Wada T., Kawasaki T., Morikawa D., Maruyama Y., Kaneko K. (2020). Supraspinatus Muscle and Tendon Stiffness Changes After Arthroscopic Rotator Cuff Repair: A Shear Wave Elastography Assessment. J. Orthop. Res..

[24] Heller G.Z., Manuguerra M., Chow R. (2016). How to analyse the Visual Analogue Scale: Myths, truths and clinical relevance. Scand. J. Pain.

[25] Vrotsou K., Ávila M., Machón M., Mateo-Abad M., Pardo Y., Garin O., Zaror C., González N., Escobar A., Cuéllar R. (2018). Constant-Murley Score: Systematic review and standardised evaluation in different shoulder pathologies. Qual. Life Res..

[26] Ibrahim M.I., Donatelli R.A., Schmitz C., Hellman M.A., Buxbaum F. (2010). Chronic Plantar Fasciitis Treated with Two Sessions of Radial Extracorporeal Shock Wave Therapy. Foot Ankle Int..

[27] Santilli G., Vetrano M., Mangone M., Agostini F., Bernetti A., Coraci D., Paoloni M., de Sire A., Paolucci T., Latini E. (2024). Predictive Prognostic Factors in Non-Calcific Supraspinatus Tendinopathy Treated with Focused Extracorporeal Shock Wave Therapy: An Artificial Neural Network Approach. Life.

[28] Louwerens J.K., Veltman E.S., van Noort A., van den Bekerom M.P. (2016). The Effectiveness of High-Energy Extracorporeal Shockwave Therapy Versus Ultrasound-Guided Needling Versus Arthroscopic Surgery in the Management of Chronic Calcific Rotator Cuff Tendinopathy: A Systematic Review. Arthrosc. J. Arthrosc. Relat. Surg..

[29] Bannuru R.R., Flavin N.E., Vaysbrot E., Harvey W., McAlindon T. (2014). High-energy extracorporeal shockwave therapy for treating chronic calcific tendinitis of the shoulder: A systematic review. Ann Intern Med.

[30] van Rijn R.M., Huisstede B.M., Koes B.W., Burdorf A., Bw K. (2010). Associations between work-related factors and specific disorders of the shoulder—A systematic review of the literature. Scand. J. Work Environ. Health.

[31] Paolucci T., Agostini F., Conti M., Cazzolla S., Mussomeli E., Santilli G., Poso F., Bernetti A., Paoloni M., Mangone M. (2023). Comparison of Early versus Traditional Rehabilitation Protocol after Rotator Cuff Repair: An Umbrella-Review. J. Clin. Med..

[32] Coraci D., Maccarone M.C., Ragazzo L., Tognolo L., Restivo D.A., Santilli G., Moreira A.L., Ferrara P.E., Ronconi G., Masiero S. (2024). Botulinum toxin in the rehabilitation of painful syndromes: Multiperspective literature analysis, lexical analysis and systematic review of randomized controlled trials. Eur. J. Transl. Myol..

[33] Agostini F., de Sire A., Bernetti A., Farì G., Damiani C., Santilli G., Alessio G., Ammendolia A., Paoloni M., Mangone M. (2023). Effectiveness of Kinesiotaping and McConnell taping combined with physical exercise on gait biomechanics in patients with patellofemoral syndrome: Non-randomized clinical trial. Clin. Ter..

[34] Agostini F., Bernetti A., Santilli G., Damiani C., Santilli V., Paoloni M., Mangone M. (2023). Efficacy of ultrasound therapy combined with cryotherapy in pain management and rehabilitation in patients with Achilles tendinopathy: A retrospective observational study. Clin. Ter..

[35] Agostini F., Bernetti A., Santilli G., Paoloni M., Santilli V., Mangone M. (2022). Laser and thermal therapy in athletes’ tennis elbow: An observational study. Med. Sport.

[36] Li W., Zhang S.X., Yang Q.I., Li B.L., Meng Q.G., Guo Z.G. (2017). Effect of extracorporeal shockwave therapy for treating patients with chronic rotator cuff tendonitis. Medicine.

[37] Testa G., Vescio A., Perez S., Consoli A., Costarella L., Sessa G., Pavone V. (2020). Extracorporeal Shockwave Therapy Treatment in Upper Limb Diseases: A Systematic Review. J. Clin. Med..

[38] Delia C., Santilli G., Colonna V., Di Stasi V., Latini E., Ciccarelli A., Taurone S., Franchitto A., Santoboni F., Trischitta D. (2024). Focal Versus Combined Focal Plus Radial Extracorporeal Shockwave Therapy in Lateral Elbow Tendinopathy: A Retrospective Study. J. Funct. Morphol. Kinesiol..

[39] Santilli G., Ioppolo F., Mangone M., Agostini F., Bernetti A., Forleo S., Cazzolla S., Mannino A.C., Fricano A., Franchitto A. (2024). High Versus Low-Energy Extracorporeal Shockwave Therapy for Chronic Lateral Epicondylitis: A Retrospective Study. J. Funct. Morphol. Kinesiol..

[40] Galasso O., Amelio E., Riccelli D.A., Gasparini G. (2012). Short-term outcomes of extracorporeal shock wave therapy for the treatment of chronic non-calcific tendinopathy of the supraspinatus: A double-blind, randomized, placebo-controlled trial. BMC Musculoskelet. Disord..

[41] Hutchinson J.L., Gusberti D., Saab G. (2019). Changing appearance of intraosseous calcific tendinitis in the shoulder with time: A case report. Radiol. Case Rep..

[42] Klauser A.S., Miyamoto H., Tamegger M., Faschingbauer R., Moriggl B., Klima G., Feuchtner G.M., Kastlunger M., Jaschke W.R. (2013). Achilles Tendon Assessed with Sonoelastography: Histologic Agreement. Radiology.

[43] Ioppolo F., Tattoli M., Di Sante L., Attanasi C., Venditto T., Servidio M., Cacchio A., Santilli V. (2012). Extracorporeal shockwave therapy for supraspinatus calcifying tendinitis: A randomised clinical trial comparing two different energy levels. Phys. Ther..

[44] Carosi M., Galeoto G., Di Gennaro S., Berardi A., Valente D., Servadio A. (2020). Transcultural reliability and validity of an Italian language version of the Constant-Murley Score. J. Orthop. Trauma Rehabilitation.

[45] Constant C., Murley A. (1987). A clinical method for functional assessment of the shoulder. Clin. Orthop. Relat. Red..

[46] Michener L.A., Yesilyaprak S.S.S., Seitz A.L., Timmons M.K., Walsworth M.K. (2013). Supraspinatus tendon and subacromial space parameters measured on ultrasonographic imaging in subacromial impingement syndrome. Knee Surg. Sports Traumatol. Arthrosc..

[47] Mifsud T., Gatt A., Micallef-Stafrace K., Chockalingam N., Padhiar N. (2023). Elastography in the assessment of the Achilles tendon: A systematic review of measurement properties. J. Foot Ankle Res..

[48] Riaz S., Sattar A., Seemal P., Majeed R., Naveed A., Abid N., Bashir S. (2023). Comparison of Extracorporeal Shockwave and High-Intensity Laser in Treating Chronic Plantar Fasciitis. Pak. J. Med. Health Sci..

[49] Bird S.B., Dickson E.W. (2001). Clinically significant changes in pain along the visual analog scale. Ann. Emerg. Med..

[50] Ratner B. (2009). The correlation coefficient: Its values range between +1/−1, or do they?. J. Target. Meas. Anal. Mark..

[51] Chen X.-M., Cui L.-G., He P., Shen W.-W., Qian Y.-J., Wang J.-R. (2013). Shear Wave Elastographic Characterization of Normal and Torn Achilles Tendons: A pilot study. J. Ultrasound Med..

[52] Hackett L., Aveledo R., Lam P.H., Murrell G.A. (2020). Reliability of shear wave elastography ultrasound to assess the supraspinatus tendon: An intra and inter-rater in vivo study. Shoulder Elb..

[53] Minafra P., Bortolotto C., Rampinini E., Calliada F., Monetti G. (2017). Quantitative Elastosonography of the Myotendinous Junction: Normal Behavior and Correlation With a Standard Measurement System During Functional Tests. J. Ultrasound Med..

[54] Ahn K.-S., Kang C.H., Hong S.-J., Jeong W.-K. (2014). Ultrasound Elastography of Lateral Epicondylosis: Clinical Feasibility of Quantitative Elastographic Measurements. Am. J. Roentgenol..

[55] Hall T.J. (2003). AAPM/RSNA Physics Tutorial for Residents: Topics in US: Beyond the basics: Elasticity imaging with US. Radiographics.

[56] Elsayed M., Hafez M.R.M., Ibrahim M.A.H. (2022). Ultrasound with shear wave elastography in diagnosis and follow-up of common extensor tendinopathy in cases with lateral epicondylitis: A cross-sectional analytic study. Egypt. J. Radiol. Nucl. Med..

[57] Rosenbaum A.J., DiPreta J.A., Misener D. (2014). Plantar Heel Pain. Med. Clin. N. Am..

[58] Cook J.L., Purdam C.R. (2009). Is tendon pathology a continuum? A pathology model to explain the clinical presentation of load-induced tendinopathy. Br. J. Sports Med..

[59] Snider M., Clancy W., Mcbeath A. (1983). Plantar fascia release for chronic plantar fasciitis in runners. Am. J. Sports Med..

[60] Dubé M.-O., Ingwersen K.G., Roy J.-S., Desmeules F., Lewis J., Juul-Kristensen B., Vobbe J., Jensen S.L., McCreesh K. (2024). Do therapeutic exercises impact supraspinatus tendon thickness? Secondary analyses of the combined dataset from two randomised controlled trials in patients with rotator cuff-related shoulder pain. J. Shoulder Elb. Surg..

[61] Torstensen T.A., Meen H.D., Stiris M. (1994). The Effect of Medical Exercise Therapy on a Patient With Chronic Supraspinatus Tendinitis. Diagnostic Ultrasound—Tissue Regeneration: A Case Study. J. Orthop. Sports Phys. Ther..

[62] De Zordo T., Fink C., Feuchtner G.M., Smekal V., Reindl M., Klauser A.S. (2009). Real-Time Sonoelastography Findings in Healthy Achilles Tendons. Am. J. Roentgenol..

[63] Dirrichs T., Quack V., Gatz M., Tingart M., Kuhl C.K., Schrading S. (2016). Shear Wave Elastography (SWE) for the Evaluation of Patients with Tendinopathies. Acad. Radiol..

[64] Aubry S., Nueffer J.-P., Tanter M., Becce F., Vidal C., Michel F. (2015). Viscoelasticity in Achilles Tendonopathy: Quantitative Assessment by Using Real-time Shear-Wave Elastography. Radiology.

[65] Kayser R., Mahlfeld K., E Heyde C. (2005). Partial rupture of the proximal Achilles tendon: A differential diagnostic problem in ultrasound imaging. Br. J. Sports Med..

[66] Hróbjartsson A., Gøtzsche P.C. (2001). Is the Placebo Powerless? An analysis of clinical trials comparing placebo with no treatment. N. Engl. J. Med..

[67] Vetrano M., D’alessandro F., Torrisi M.R., Ferretti A., Vulpiani M.C., Visco V. (2011). Extracorporeal shock wave therapy promotes cell proliferation and collagen synthesis of primary cultured human tenocytes. Knee Surg. Sports Traumatol. Arthrosc..

[68] Wang C.-J., Huang H.-Y., Pai C.-H. (2002). Shock wave-enhanced neovascularization at the tendon-bone junction: An experiment in dogs. J. Foot Ankle Surg..

[69] Wang F.S., Yang K.D., Chen R.F., Wang C.J., Sheen-Chen S.M. (2002). Extracorporeal shock wave promotes growth and differentiation of bone-marrow stromal cells towards osteoprogenitors associated with induction of TGF-β1. J. Bone Jt. Surg. Br. Vol..

[70] Di Meglio F., Sacco A.M., Belviso I., Romano V., Sirico F., Loiacono C., Palermi S., Pempinello C., Montagnani S., Nurzynska D. (2020). Influence of Supplements and Drugs used for the Treatment of Musculoskeletal Disorders on Adult Human Tendon-Derived Stem Cells. Muscles Ligaments Tendons J..

[71] Palermi S., Gnasso R., Belviso I., Iommazzo I., Vecchiato M., Marchini A., Corsini A., Vittadini F., Demeco A., De Luca M. (2023). Stem cell therapy in sports medicine: Current applications, challenges and future perspectives. J. Basic Clin. Physiol. Pharmacol..

[72] Prado-Costa R., Rebelo J., Monteiro-Barroso J., Preto A.S. (2018). Ultrasound elastography: Compression elastography and shear-wave elastography in the assessment of tendon injury. Insights Imaging.

[73] Sharp S.J., Poulaliou M., Thompson S.G., White I.R., Wood A.M. (2014). A Review of Published Analyses of Case-Cohort Studies and Recommendations for Future Reporting. PLoS ONE.

[74] Price J.H., Murnan J. (2004). Research Limitations and the Necessity of Reporting Them. Am. J. Health Educ..

[75] Santilli G., Mangone M., Agostini F., Paoloni M., Bernetti A., Diko A., Tognolo L., Coraci D., Vigevano F., Vetrano M. (2024). Evaluation of Rehabilitation Outcomes in Patients with Chronic Neurological Health Conditions Using a Machine Learning Approach. J. Funct. Morphol. Kinesiol..

[76] Oh J.H., Kim S.H., Kang J.Y., Oh C.H., Gong H.S. (2010). Effect of Age on Functional and Structural Outcome after Rotator Cuff Repair. Am. J. Sports Med..

